# Exciton quantum confinement in nanocones formed on a surface of CdZnTe solid solution by laser radiation

**DOI:** 10.1186/1556-276X-7-514

**Published:** 2012-09-20

**Authors:** Artur Medvid', Natalia Litovchenko, Aleksandr Mychko, Yuriy Naseka

**Affiliations:** 1 Riga Technical University, Azenes Street 14, Riga, 1048, Latvia; 2 Institute of Semiconductor Physics, NAS of Ukraine, Pr. Nauki 28, Kyiv, 03028, Ukraine

**Keywords:** Nanocones, Exciton quantum confinement effect, Thermogradient effect, CdZnTe, Nd:YAG laser

## Abstract

The investigation of surface morphology using atomic force microscope has shown self-organizing of the nanocones on the surface of CdZnTe crystal after irradiation by strongly absorbed Nd:YAG laser irradiation at an intensity of 12.0 MW/cm^2^. The formation of nanocones is explained by the presence of a thermogradient effect in the semiconductor. The appearance of a new exciton band has been observed after irradiation by the laser which is explained by the exciton quantum confinement effect in nanocones.

## Background

Nowadays, nanostructures are one of the most investigated objects in semiconductor physics, especially the quantum confinement effect (QCE) in such quantum systems as quantum dots or 0D [1-5], quantum wires or 1D [6-8] and quantum wells or 2D [9-14]. In the case of nanostructures, the energy band diagram of the semiconductor is strongly changed. This leads to a crucial change of semiconductor properties, such as electrical properties (the change of free charge carriers concentration and their mobility), optical properties (absorption coefficient, reflectivity coefficient, and radiative recombination efficiency), and mechanical and thermal properties [15]. Another possibility to change a property of a semiconductor is by using solid solution, such as Cd_1−*x*_Zn_*x*_Te [16] and Si_1−*x*_Ge_*x*_[17], which change the component content. It was shown [18] that the shapes and sizes of the mentioned quantum systems have more influence on the properties of a semiconductor than its component content. For example, the ‘blue shift’ of Si_0.7_Ge_0.3_ photoluminescence (PL) spectrum of nanocones is up to 1.2 eV, but the possible maximal shift of PL spectra due to change of *x* is only up to 0.33 eV. Moreover, the band of PL spectrum is broader and more intense due to QCE and graded band gap. Moreover, nanocones enhance radiation hardness of CdZnTe detector, as shown in [19].

In this paper, we report about the appearance of a new band in PL spectra of Cd_1−*x*_Zn_*x*_Te solid solution irradiated by Nd:YAG laser, which is explained by exciton QCE in nanocones formed on the irradiated surface of the sample.

## Methods

The laser processing was performed on the samples of Cd_1−*x*_Zn_*x*_Te solid solution with *x* = 0.1 in ambient atmosphere at room temperature, pressure 1 atm, and 80% humidity. The surface of Cd_1−*x*_Zn_*x*_Te sample was irradiated by pulses of Nd:YAG laser with the wavelength of *λ* = 532 nm, pulse duration of *τ* = 15 ns and power *p* = 1 MW. The spot of laser beam with 3 mm diameter was moved by 20 μm steps over the surface of the sample. Atomic force microscope (AFM) was used for the study of the irradiated surface morphology. The low-temperature PL at 5 K was carried out to investigate the optical properties of the nanostructures formed by laser radiation (LR) on the samples. He-Ne laser with λ = 632.8 nm was used as an excitation source.

## Results and discussion

**Figure 1:**
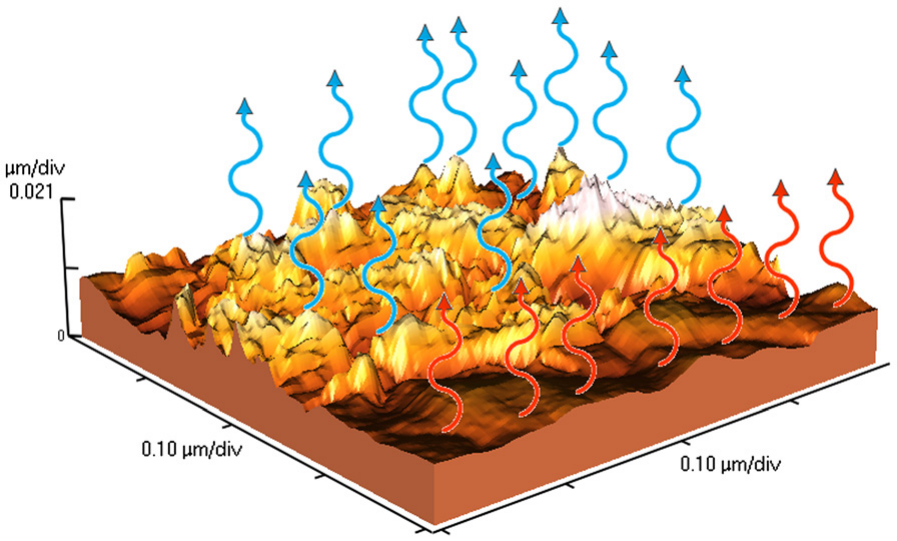
**Atomic force microscope 3D image of the Cd**_**1−*x***_**Zn**_***x***_**Te (*x*** **= 0.1) surfaces after irradiation by the laser at intensity of 12 MW/cm**^**2**^**.** The blue and red curly arrows show the different surfaces with different luminescence properties.

The formation of nanocones on a surface of Cd_1−*x*_Zn_*x*_Te solid solution with *x* = 0.1 after irradiation by the strongly absorbed Nd:YAG laser radiation with intensity of *I* = 12.0 MW/cm^2^ was observed using AFM, as shown in Figure 1.

**Figure 2:**
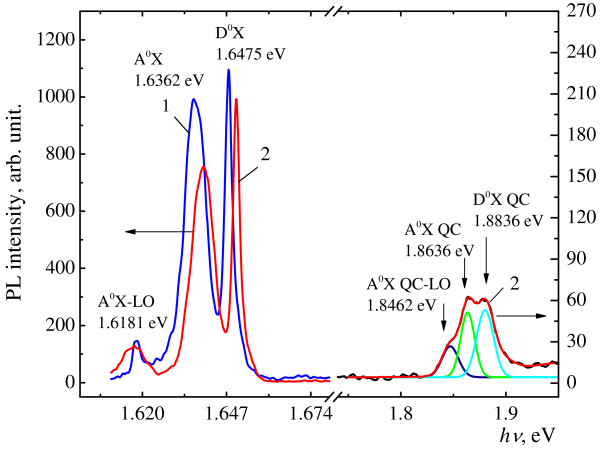
**Photoluminescence spectra of the Cd**_**1−*x***_**Zn**_***x***_**Te (*x*** **= 0.1) measured at temperature of 5 K before (curve 1) and after (curve 2) the irradiation by the laser at *I*** **= 10.0 MW/cm**^**2**^**.**

While studying the optical properties of nanocones, a new exciton band at energy up to 1.87 eV in PL spectrum for the first time was found, as shown in Figure 2. The PL spectrum is rather complex, consisting of an intense line (A^0^, X) at 1.6362 eV, which is ascribed to bound excitons to shallow acceptors (Cd vacancies, *V*_Cd_) and its longitudinal optical (LO)-phonon replica at 1.6181 eV, an intense line (D^0^, X) at 1.6475 eV ascribed to bound excitons to shallow donors (Cd interstitial atoms, *I*_Cd_). At the same time, the shift of A^0^X and D^0^X exciton lines of 3.2 and 2.7 meV, correspondingly, toward the higher energy of quantum, that is, the so-called ‘blue shift’ was observed, as shown in Figure 2. The appearance of a new PL band is explained by exciton quantum confinement (EQC) effect in nanocones, and the blue shift of A^0^X and D^0^X exciton bands - due to photo-induced mechanical compressive stress of the top layer.

This process takes place in the following way: the irradiation of the Cd_1−*x*_Zn_*x*_Te solid solution by the laser leads to the drift of Cd atoms toward the irradiated surface and of Zn atoms - in the opposite direction due to high gradient of temperature. This is so-called thermogradient effect (TGE) [20]. As a result, the formation of CdTe/Cd_1−*x*1_Zn_*x*1_Te heterostructure, where *x*_1_ > *x*, takes place due to the replacement of Zn atoms by Cd atoms at the irradiated surface. At the same time, the opposite process takes place under the top layer. In the buried layer of the semiconductor, Zn atoms replace Cd atoms. At least three factors determine A^0^X and D^0^X exciton lines position in PL spectrum. They are as follows: the concentration of Zn atoms in the CdTe top layer and in CdZnTe buried layer, 2D EQC effect in the CdTe layer when its thickness is comparable with Bohr radius of the exciton, and the mechanical compressive stress of the CdTe top layer due to mismatch of CdTe and CdZnTe crystalline lattice constant.

**Figure 3:**
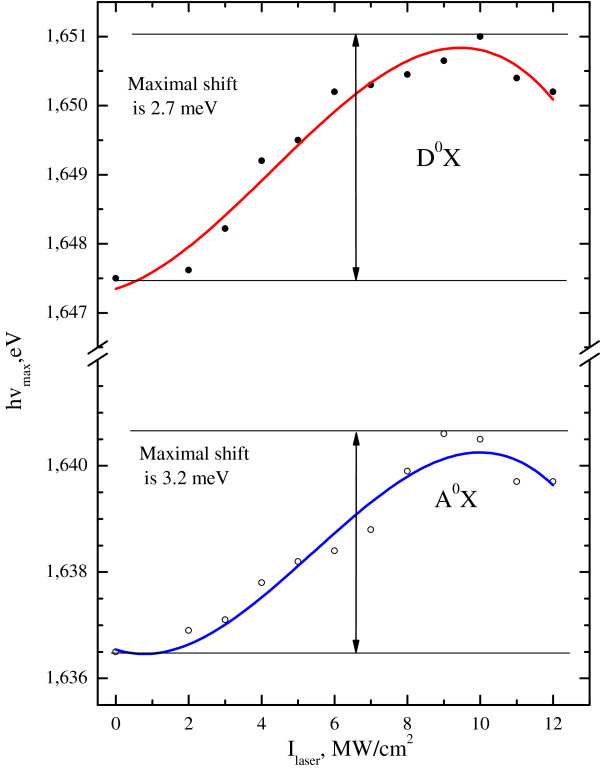
**Blue shift of A**^**0**^**X and D**^**0**^**X exciton lines in PL spectra as function of the laser intensity.**

**Figure 4:**
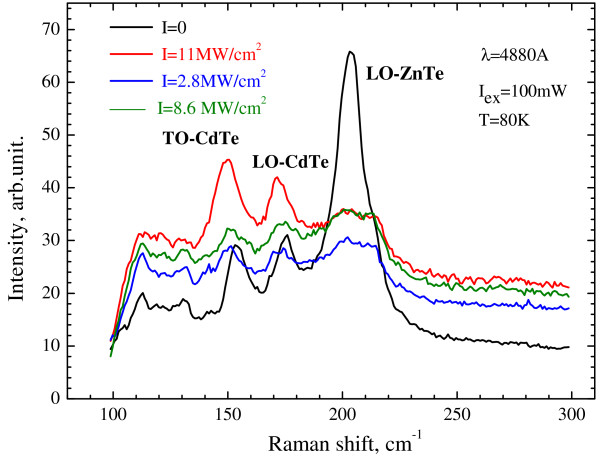
**Raman back scattering spectra of nonirradiated and irradiated CdZnTe sample.** Intensities of LR are as follows: *I* = 0, black curve; *I* = 2.8 MW/cm^2^, blue curve; *I* = 8.6 MW/cm^2^, green curve; and *I* = 11.0 MW/cm^2^, red curve.

The decrease of Zn atoms concentration in the top layer with increased intensity of LR, according to the proposed model, leads to the ‘red shift’ of the exciton bands in PL spectra, as was shown in [21], but increase of the Zn atoms concentration in the buried CdZnTe layer manifests itself in the blue shift of PL spectrum, as shown in Figure 3. These effects do not compensate each other because they take place in different layers. This unusual situation can be explained by different input of these layers in the position and intensity of PL spectrum. If the top layer is excited by short wavelength light, then mostly the red shift of PL spectrum will be observed; but if mainly the buried layer is excited, for example, due to small thickness or transparency of top layer, then the blue shift will be observed. Of course, it is possible to observe both PL spectra simultaneously at intermediate situation. Such situation is exactly observed in the PL spectrum in Figure 2, after the destruction of the CdTe top layer and formation of nanocones on the irradiated surface of the sample. The relaxation of the mechanical compressive stress in CdTe layer corresponds to the decreasing part of the curves in Figure 3. It is manifested as the self-assembly of nanocones on the irradiated surface of the structure as explained by Stransky-Krastanov' growth mode. A simultaneous appearance of a new exciton band at 1.872 eV in PL spectrum at high intensity of LR takes place. The reconstruction of this band according to Gaussian fitting shows that it consists of three lines which look like A^0^X, D^0^X, and A^0^X-LO lines (the distance between the lines and their full width at half maximum are the same) in the nonirradiated PL spectrum of the semiconductor. Therefore, we connect the appearance of the new lines in PL spectrum with the nanocones' formation on the irradiated surface of the semiconductor and with EQC in the nanocones. We denote them as A^0^XQC and D^0^XQC lines. An evidence of the mechanical stress relaxation process in CdTe layer is a non-monotonic dependence of the blue shift as a function of LR intensity, as shown in Figure 3. Such non-monotonic dependence have been observed in p- and n-type Si after studying of mechanical micro hardness of Si after irradiation by Nd:YAG laser [22]. The calculation of the mechanical compressive stress in CdTe top layer using the maximum of the blue shift of A^0^X exciton line from Figure 3 and dEg/dP = 10 eV/GPa [23], where Eg and P are band gap of CdTe crystal and mechanical stress, correspondingly, gives *P* = 4.62 × 10^5^ Pa. This value corresponds to the ultimate strength limit of CdTe [24]. The calculation of the quantum dot diameter using formula from [25] and the blue shift of A^0^XQC in the PL spectrum of 0.27 eV gives about 10.0 nm diameter of the quantum dots. These data correspond to the size of nanocones [26] (height and diameter of the bottom of the cones are 10.0 nm) measured using 3D image of AFM as can be seen in Figure 1. An evidence of the presence of EQC in nanocones is the decrease of LO phonon energy by 0.7 meV in the PL spectrum. This is the so-called ‘phonon quantum confinement effect’ [27]. Moreover, the increase of Huang-Rhys factor for A^0^X-LO line up to three times is a good evidence of EQC effect in the nanocones. Our calculation of Zn atoms distribution depending on the intensity of LR using the thermo-diffusion equation has shown that the process of CdTe/Cd_1−*x*1_Zn_*x*1_Te heterostructure formation is characterized by the gradual increase of Zn atoms concentration in the buried layer with intensity of LR up to 8% (*x*_1_ = 0.18). The thickness of the CdTe layer after irradiation by the laser with intensity of *I* = 12.0 MW/cm^2^ becomes 10 nm. The process of nanocones formation is characterized by the LR threshold intensity of approximately *I* = 10.0 MW/cm^2^, as can be seen in Figure 3: nanocones formation starts at the maximums of the blue shift position. Therefore, the process of the nanocones formation is characterized by two stages: the first stage is the formation of CdTe/CdZnTe heterostructure, and the second stage is the nanocones’ self-assembly due to laser annealing of the mechanical compressive stress in CdTe layer. An evidence of the presence of the first stage of nanocones formation process is the redistribution of the intensity of LO-ZnTe, TO-CdTe, and LO-CdTe phonon bands in Raman back scattering spectra, as shown in Figure 4.

It means that before the irradiation of the sample by the laser, the intensity of LO-ZnTe phonon band was three to four times higher than the intensity of TO and LO-CdTe phonon bands, but after irradiation the opposite situation in Raman spectra is observed.

## Conclusions

The studies of the effect of highly absorbed laser radiation on the optical properties of the Cd_1−*x*_Zn_*x*_Te (*x* = 0.1) compound have revealed the formation of nanocones on the surface of the semiconductor under irradiation by the Nd:YAG laser within the intensity range of 9.0-12.0 MW/cm^2^ and the simultaneous appearance of a new PL band at 1.87 eV, which is explained by the exciton quantum confinement effect in nanocones.

The TGE has the main role in redistribution of Zn atoms at the surface of Cd_1−*x*_Zn_*x*_Te irradiated by the second harmonic of Nd:YAG laser. The graded band gap structure with open optical window is formed on the top of nanocones after the irradiation by Nd:YAG laser at the intensity 4.0-12.0 MW/cm^2^. Formation of a graded band gap with a close of optical window in Cd_1−*x*_Zn_*x*_Te crystal is possible under irradiation by the second harmonic of Nd:YAG laser at the intensity 0.2-2.0 MW/cm^2^. A two-stage model of the nanocones formation on the surface of the Cd_1−*x*_Zn_*x*_Te (*x* = 0.1) under the irradiation by Nd:YAG laser at the intensity 4.0-12.0 MW/cm^2^ was proposed.

## Abbreviations

AFM: Atomic force microscope; 0D: Quantum dots; 1D: Quantum wires; 2D: Quantum wells; EQC: Exciton quantum confinement; *I*_Cd_: Cd interstitial atoms; LR: Laser radiation; PL: Photoluminescence; QCE: Quantum confinement effect; TGE: Thermogradient effect; *V*_Cd_: Cd vacancies.

## Competing interests

The authors declare that they have no competing interests.

## Authors' contributions

AM and LN conceived the studies and coordinated the experiment. All of the authors participated to the analysis of the data and wrote the article. AMy and YN carried out the sample preparation, the measurements for solid solutions of CdZnTe. All the authors read and approved the manuscript.
